# Gene therapy-emulating small molecule treatments in cystic fibrosis airway epithelial cells and patients

**DOI:** 10.1186/s12931-019-1214-8

**Published:** 2019-12-21

**Authors:** Q. Yang, A. R. Soltis, G. Sukumar, X. Zhang, H. Caohuy, J. Freedy, C. L. Dalgard, M. D. Wilkerson, H. B. Pollard, B. S. Pollard

**Affiliations:** 1 Department of Anatomy, Physiology and Genetics, Uniformed Services University School of Medicine- America’s Medical School, Uniformed Services University of the Health Sciences (USUHS), Bethesda, MD 20814 USA; 2 Collaborative Health Initiative Research Program (CHIRP), The American Genome Center (TAGC), Uniformed Services University of the Health Sciences (USUHS), Bethesda, MD 20814 USA; 3 Silver Pharmaceuticals, Rockville, MD 20854 USA

**Keywords:** Cystic fibrosis, Inflammation, Kalydeco, Ivacaftor, Tezacaftor, Lumacaftor, Digitoxin

## Abstract

**Background:**

Several small molecule corrector and potentiator drugs have recently been licensed for Cystic Fibrosis (CF) therapy. However, other aspects of the disease, especially inflammation, are less effectively treated by these drugs. We hypothesized that small molecule drugs could function either alone or as an adjuvant to licensed therapies to treat these aspects of the disease, perhaps emulating the effects of gene therapy in CF cells. The cardiac glycoside digitoxin, which has been shown to inhibit TNFα/NFκB signaling in CF lung epithelial cells, may serve as such a therapy.

**Methods:**

IB3–1 CF lung epithelial cells were treated with different Vertex (VX) drugs, digitoxin, and various drug mixtures, and ELISA assays were used to assess suppression of baseline and TNFα-activated secretion of cytokines and chemokines. Transcriptional responses to these drugs were assessed by RNA-seq and compared with gene expression in AAV-[*wildtype*]CFTR-treated IB3–1 (S9) cells. We also compared in vitro gene expression signatures with in vivo data from biopsied nasal epithelial cells from digitoxin-treated CF patients.

**Results:**

CF cells exposed to digitoxin exhibited significant suppression of both TNFα/NFκB signaling and downstream secretion of IL-8, IL-6 and GM-CSF, with or without co-treatment with VX drugs. No evidence of drug-drug interference was observed. RNA-seq analysis showed that gene therapy-treated CF lung cells induced changes in 3134 genes. Among these, 32.6% were altered by digitoxin treatment in the same direction. Shared functional gene ontology themes for genes suppressed by both digitoxin and gene therapy included inflammation (84 gene signature), and cell-cell interactions and fibrosis (49 gene signature), while genes elevated by both were enriched for epithelial differentiation (82 gene signature). A new analysis of mRNA data from digitoxin-treated CF patients showed consistent trends in expression for genes in these signatures.

**Conclusions:**

Adjuvant gene therapy-emulating activities of digitoxin may contribute to enhancing the efficacy of currently licensed correctors and potentiators in CF patients.

## Introduction

Cystic fibrosis (CF) is a life-limiting autosomal recessive genetic disease due to mutations in the *CFTR* gene [1–3]. In the lung, the common CF mutation [*F508del*] [4, 5], and the less common [*G551D*] mutation [6–9], are both associated with a hyper-proinflammatory phenotype that is due to constitutive activation of the TNFα/NFκB signaling pathway [10–14]. For both mutations, failure to suppress inflammation leads to further reactive inflammation, exacerbation of constitutive secretion of mucin, local airway obstruction and hypoxia, infection, and, finally, complete loss of lung function. Recently, to treat the pulmonary manifestations of CF, a “potentiator” drug, VX-770, has been licensed that induces chloride channel activity in the [*G551D*] gating mutant [15]. A modestly effective “corrector” drug, VX-809, has been licensed to treat the common [*F508del*] mutation in combination with VX- 770 [16]. More recently, a licensed combination of the corrector drug VX-661 with VX-770 has been shown to be safer and possibly more effective in CF patients homozygous [17] and heterozygous [18] for the [*F508del*] CFTR mutation. However, despite substantial and successful responses in CF patients, there remain other aspects of the disease for which the corrector and potentiator drugs are less effective [19–22]. The need to fill this gap is illustrated by the Cystic Fibrosis Foundation (CFF)‘s recent offer of support for “discovery of ways to dampen the exaggerated immune response that causes chronic inflammation without affecting the body’s natural defenses against infection” [23].

It is possible that efficacious, adjuvant anti-inflammatory drugs could be combined therapeutically with currently licensed drugs to ameliorate even severe CF disease phenotypes [24]. Unfortunately, several CF anti-inflammatory adjuvant drugs, including ibuprofen and prednisone, carry significant risk to patients [25–29]. However, there are also several new candidate anti-inflammatory drugs in early stages of development [30–32]. Among these new candidates is the cardiac glycoside drug digitoxin. It was first tested in this context in CF lung epithelial cells*,* where it was found to inhibit TNFα/NFκB signaling and downstream IL-8 secretion [33, 34]. This discovery led to testing digitoxin in CF patients as an anti-inflammatory agent in a Phase 2, dose escalation, placebo-controlled clinical trial (NCT00782288, clinicaltrials.gov). It was found that mono-therapy digitoxin not only suppressed respiratory adverse events by 69% (*p* = 0.0365), but also blocked TNFα/NFκB signaling and *IL-8* gene expression in nasal epithelial cells biopsied from drug-treated CF patients [35]. The fact that digitoxin could act as an anti-inflammatory agent in vivo, motivated us to consider the use of digitoxin as a CF treatment, either alone or as an adjuvant to licensed VX drugs. However, we reasoned that it was critical to carefully test combinations of digitoxin and VX drugs therapy in vitro, in anticipation of adjuvant use in patients, because drug-drug interferences have been described for VX-809 and VX-770 [36]**,** as well as between VX-770 and CFTR itself [37].

Here, we show that digitoxin is able to *suppress* proinflammatory TNFα/NFκB signaling and downstream secretion of IL-8, IL-6 and GM-CSF, when tested alone or in the presence of individual or combinations of licensed corrector and potentiator drugs. By contrast, the VX-drugs are relatively inactive in terms of inhibiting chemokine and cytokine secretion. Expression changes measured by RNA-seq are consistent with this conclusion, and also show that digitoxin, alone or in combination with VX-drugs, causes changes that significantly emulate some of the effects of AAV-[*wildtype*]CFTR gene therapy in CF lung epithelial cells. Finally, re-examination of nasal epithelial cell mRNA expression data from CF patients treated with digitoxin suggests that there are significant clinical parallels with the in vitro data collected in this study. Taken together, these data suggest adjuvant gene therapy-emulating activities of digitoxin may contribute to enhancing the efficacy of currently licensed correctors and potentiators in CF patients.

## Materials and methods

General information is given below. Greater detail is included in Additional file 1.

### Cells and drugs

CF lung epithelial IB3–1 cells ([*F508del/W1282X*]), and daughter IB3–1/S9 cells stably transfected with AAV-[*wildtype*] CFTR [38], were grown in serum-free LHC-8 medium (Biofluids, Bethesda, MD), formulated without gentamycin, *exactly* as previously described [33]. Digitoxin was obtained from Sigma (Sigma-Aldrich Corp., St. Louis, MO), and dissolved in 100% ethanol prior to dilution in PBS. The final ethanol concentration was 0.001-0.0025%. VX-770, VX-809 and VX-661 were obtained from Selleckchem.com (Houston, TX), and dissolved in 100% DMSO prior to dilution in PBS.

Fisher rat thyroid (FRT) cells stably expressing human [*G551D*]CFTR were generously provided by Dr. Eric Sorscher (Emory University, Atlanta, GA) and grown in F-12 Modified Coon’s medium supplemented with 10% FBS, 200 μg/ml hygromycin, 0.23% sodium bicarbonate, 100 units/ml penicillin and 100 μg/ml streptomycin. FRT cells were seeded onto Costar 0.4-mm Snapwell inserts, cultured for 5 days as electrically resistive monolayers, and then treated for 24 h at 37 °C with digitoxin (25 nM) ± VX-770 (3 μM).

### Reporter gene assays

IB3–1 cells were seeded in 6 well plates overnight, then subsequently cotransfected overnight (16 h) with *NFκB-luc* and *lacZ* plasmids using Lipofectamine 3000 transfection reagent (Invitrogen). The cells were treated with 20 ng/ml TNFα and/or 25 nM digitoxin or/and VX drugs overnight. Cells were harvested and lysed with 1x passive lysis buffer. Luciferase assays were performed with the Promega Luciferase Assay System. The luciferase values were normalized to β-galactosidase activity. Differential statistics were calculated between groups using two-tailed t-tests after normalizing individual experimental values to TNFα-treated controls (*n* = 4–7 biological replicates for all groups considered).

### Measurement of cytokines and chemokines

Cytokines and chemokines were assayed on the SECTOR® S 600 instrument (Meso Scale Discovery, Gaithersburg, MD, USA). The Human Pro-Inflammatory 9 Plex Tissue Culture kit (MesoScale catalog #K15007B-2) was used for the measurement of IL-2, IL-8, IL-12p70, IL-1β, GM-CSF, IFN-γ, IL-6, IL-10, and TNF-α [39]. Differential statistics were calculated between groups using two-tailed t-tests after normalzing individual experimental values to TNFα-treated controls (*n* = 4–7 biological replicates for all groups considered).

### Western blot analysis

IB3–1 cells were lysed in M2 buffer (20 mM pH 7.0 Tris, 0.5% NP-40, 250 mM NaCl, 3 mM EDTA, 3 mM EGTA, 2 mM dithiothreitol, 0.5 mM phenylmethylsulfonyl fluoride, 20 mM β-glycerol phosphate, 10 mM, 4-nitrophenyl phosphate disodium salt, 1 mM sodium vanadate, 1 mg/ml of leupeptin). 20 μg of the cell lysate protein from each sample were fractionated by SDS-PAGE and immunoblotted. Blots were visualized with chemiluminescent substrate (Millipore, Rockville, MD) and band densities were measured using the NIH ImageJ program [40]. Anti-IκBα antibody was from Cell Signaling Technologies (Boston, MA). The antibody for β-actin was from Sigma (Sigma-Aldrich, St. Louis, MO). Differential statistics were calculated between groups using two-tailed t-tests after normalizing individual experimental values to TNFα-treated controls (*n* = 3 biological replicates). Westerns were also performed on the WES instrument (Protein Simple, San Jose, CA) according to manufacturer’s instructions.

### RNA-seq

Cultured cells were harvested in Qiazol (Qiagen, Germantown, MD) and homogenized using a QIAshredder (Qiagen) before isolation of total RNA using the RNeasy Mini Kit (Qiagen). Total RNA was quantified via a fluorescence dye-based methodology using the Quant-IT RiboGreen RNA Reagent (Thermo Scientific, Waltham, MA) and assay measurement with a Spectramax Gemini XPS plate reader (Molecular Devices, San Jose, CA).

### Bioinformatics

Details for alignment and sample quality assessment, gene quantification and differential expression analysis, gene ontology and clustering analysis, and human exon array expression analysis are given in Additional file 1**:** Materials and Methods.

## Results

### Experimental design

**Fig. 1:**
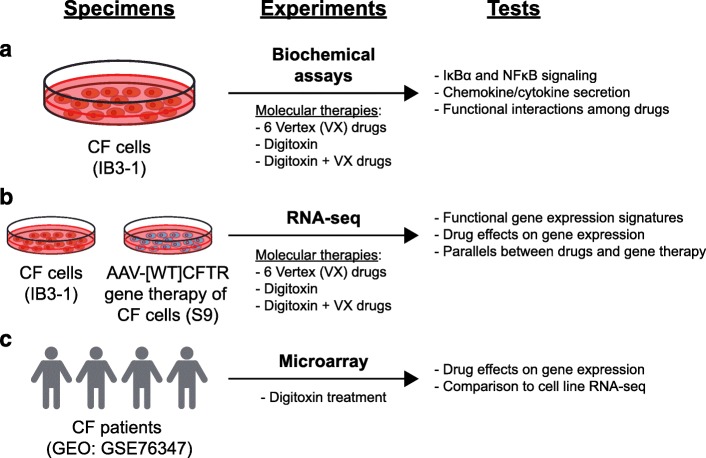
Experimental Design. **a** CF cells (IB3–1) are treated with small molecule therapies. Tests are for consequences on IκBα and NFκB signaling, chemokine and cytokine secretion, and functional interactions among drugs. **b** IB3–1 and AAV[*WT*]CFTR gene therapy (S9) cell transcriptomes are profiled with RNA-seq. Tests are for proinflammatory gene expression, drug effects on gene expression, and parallels between drugs and gene therapy. **c** Recent CF patient gene expression data is compared with in vitro data. Tests are based on re-analysis of Affymetrix microarray mRNA data from nasal epithelial biopsies of CF patients, followed by comparisons with CF cell RNA-seq data

In this study, we treated IB3–1 CF cells with various combinations of digitoxin and Vertex drugs (VX-661, VX-770, and VX-809) and interrogated IκBα/NFκB signaling, chemokine and cytokine secretion, and transcriptional responses (Fig. 1a). To gain potential therapeutic perspective on these data, we also analyzed gene therapy-corrected daughter IB3–1/S9 (“S9”) cells that were stably transfected with AAV-[*wildtype*]CFTR with RNA-seq [33] (Fig. 1b). Lastly, we interrogated in vivo digitoxin transcriptional profiles from the prior clinical trial data for gene sets uncovered in the in vitro RNA-seq data (Fig. 1c). Abbreviations are provided in Additional file 2.

### Digitoxin effects on TNFα/NFκB signaling and chemokine/cytokine secretion in CF cells

**Fig. 2:**
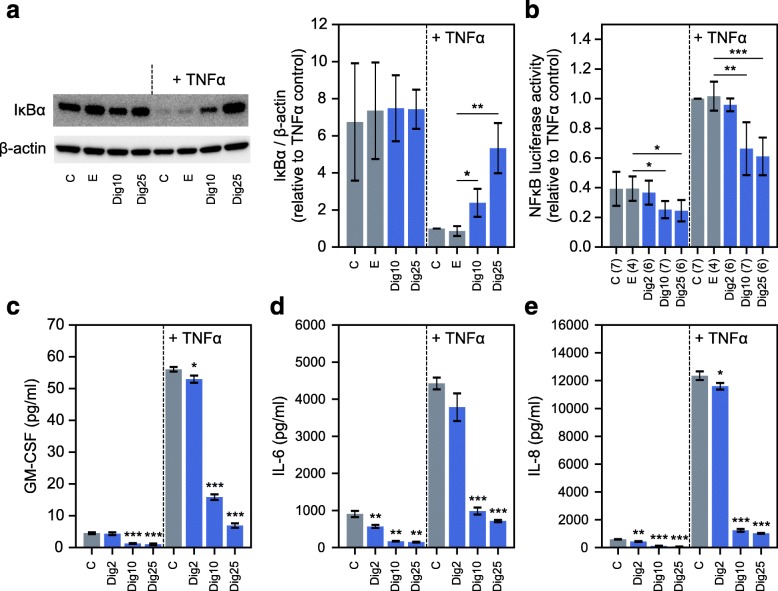
Influence of digitoxin on NFκB signaling and cytokine/chemokine secretion from CF cells. **a** Western blot experiments demonstrate that digitoxin stabilizes IκBα in the presence of TNFα (*n* = 3 biological replicates per condition; representative blot is displayed). **b** Digitoxin suppresses baseline and TNFα-activated NFκB-driven luciferase expression (numbers of biological replicates per condition are indicated in parentheses). (**C-E**) Digitoxin suppresses GM-CSF (**c**), IL-6 (**d**), and IL-8 (**e**) secretion under baseline and TNFα-activated conditions (*n* = 3; digitoxin-treated conditions are compared against respective non-TNFα-treated or TNFα-treated controls). Abbreviations: C (Control, no additions), E (ethanol), Dig2 (2 nM digitoxin), Dig10 (10 nM digitoxin), and Dig25 (25 nM digitoxin). Significant pairwise two-tailed t-test results are also displayed: * *p* < 0.05; ** *p* < 0.01; *** *p* < 0.001

Digitoxin and VX drug effects were tested on the TNFα/NFκB signaling pathway in IB3–1 CF lung epithelial cells. Western blotting experiments indicated that IκBα is detectable and stable under control conditions (Fig. 2a). By contrast, when TNFα/NFκB signaling is activated by TNFα, IκBα is profoundly destabilized. However, in the added presence of digitoxin, IκBα is dose-dependently stabilized. In addition, we observed that digitoxin dose-dependently blocks NFκB-driven luciferase activity, whether analyzed under control conditions or in the presence of added TNFα (Fig. 2b). Consistently, digitoxin dose-dependently suppresses secretion of GM-CSF, IL-6 and IL-8 under baseline and TNFα-activated conditions (Fig. 2c-e). These data are consistent with prior data showing dose-dependent suppressive effects of digitoxin on secretion of IL-8 from CF cells when incubated under baseline conditions only [33]. These experiments additionally show that at least two other TNFα/NFκB- activated mediators, IL-6 and GM-CSF, seem to be equivalently susceptible to inhibition by digitoxin treatment.

### Anti-inflammatory activities in the presence of correctors and potentiators

**Fig. 3:**
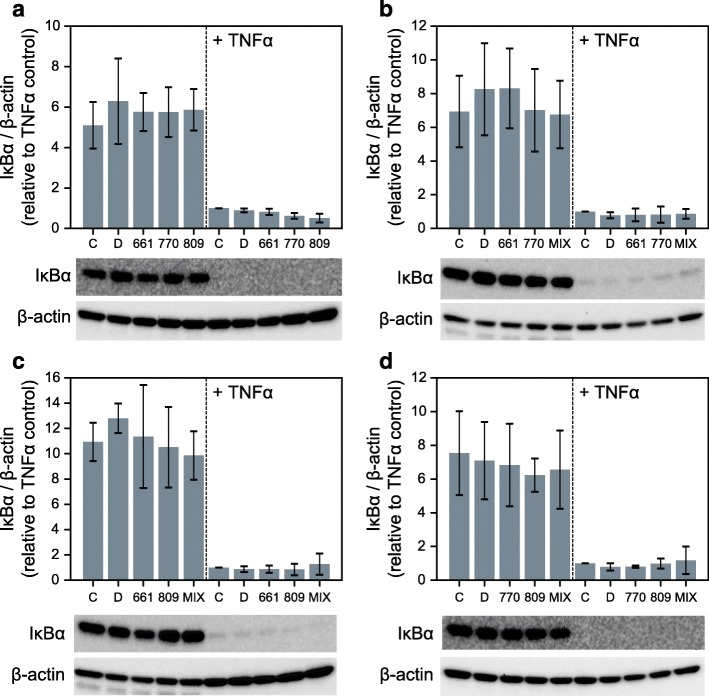
Influence of VX drugs on IκBα stabilization in CF cells. **a**-**d** Influence of VX drug monotherapies (**a**) and combination therapies (**b**-**d**) on IκBα stability without and with TNFα treatment (*n* = 3 biological replicates for all conditions; representative Western blots per experiment are displayed). Abbreviations: C (Control, no additions), D (DMSO, dimethylsufoxide), 661 (VX-661; Tezacaftor®, 3 μM), 770 (VX-770, Ivacaftor® 1 μM), 809 (VX-809, Lumacaftor®, 3 μM), MIX (mixture of VX-661 and VX-770 [**B**], VX-661 and VX-809 [**C**], or VX-770 and VX-809 [**D**])

**Fig. 4:**
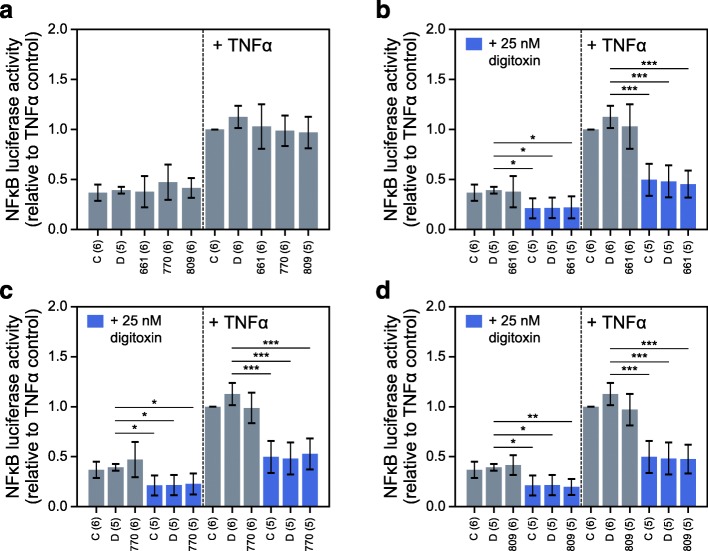
Influence of VX drug, digitoxin, and combination VX drug-digitoxin treatment on NFκB signaling in CF cells. **a** Influence of VX drug monotherapies on baseline and TNFα-activated NFκB-driven luciferase expression. **b**-**d** Influence of VX-661 (**b**), VX-770 (**c**), and VX-809 (**d**), alone (gray bars) or in combination with 25 nM digitoxin (blue bars), on baseline and TNFα-activated NFκB-driven luciferase expression. Numbers of biological replicates per condition are indicated in parentheses. Abbreviations: C (Control, no additions), D (DMSO, dimethylsufoxide), 661 (VX-661; Tezacaftor®, 3 μM), 770 (VX-770, Ivacaftor® 1 μM), 809 (VX-809, Lumicaftor®, 3 μM). Significant pairwise two-tailed t-test results are also displayed: * *p* < 0.05; ** *p* < 0.01; *** *p* < 0.001

To determine what effects, if any, the licensed VX drugs have on TNFα/NFκB signaling, we assessed IκBα protection in IB3–1 cells treated with VX-661, VX-770, and VX-809, independently (Fig. 3a) and in all pairwise combinations (Fig. 3b-d). These data show that the VX drugs have no apparent consequence on the stability of IκBα, whether tested at baseline or with TNFα activation. Consistently, the individual VX drugs also have no effect on either baseline or TNFα-activated NFκB activation (Fig. 4a). In addition, digitoxin-dependent suppression of NFκB activation is not affected by the presence of individual VX drugs (Fig. 4b-d). Furthermore, the same lack of VX drug activity is found when measuring drug effects on secretion of GM-CSF, IL-6 and IL-8 from CF cells. For example, in CF cells stimulated by TNFα, there are virtually no effects on secretion of these proinflammatory mediators**,** either by individual VX drugs (Additional file 3) or by pairwise mixtures (Additional file 4). Modest effects of VX drugs are seen in some instances on baseline secretion. However, the suppressive effects of digitoxin are still potently and significantly evident in the presence of all VX drugs. These data thus suggest not only that the VX drugs have little effect on proinflammatory signaling, but also that the presence of VX drugs do not adversely impact on the ability of digitoxin to suppress inflammation.

Finally, to directly test for possible interference by digitoxin on VX drug action, we used FRT cells transfected with [*G551D*]CFTR to measure induced chloride conductance. These experiments revealed that digitoxin modestly (but significantly) increases chloride conductance on its own, when incubated for 24 h, or when co-incubated with VX-770 (Additional file 5). Thus, digitoxin does not interfere with VX-770 function, and may somewhat augment VX-770 activity.

### Transcriptional alterations in CF lung epithelial cells following gene therapy and drug treatments

**Fig. 5:**
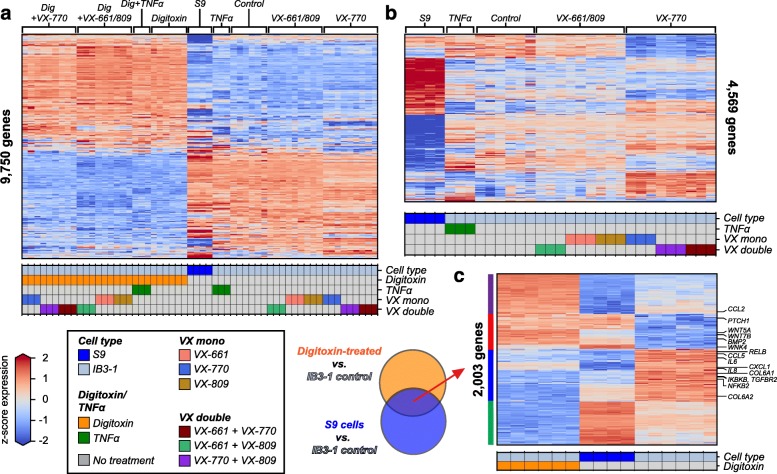
Genome-wide differential mRNA expression among drug and gene therapy treatments in CF cells. **a** Differentially expressed genes (FDR < 0.01, |log_2_ fold-change| > 0.5, mean TPM > 1) between all pairs of treatments displayed as a heatmap (9750 total). Genes are mean centered and scaled (i.e. z-scored) across conditions and are colored according to the colorbar legend (lower left, blue underexpressed, red overexpressed). Colored matrix below heatmap indicates treatment condition and cell type. **b** Differentially expressed genes between non-digitoxin treated samples, including TNFα, VX drug (mono and combination) and gene therapy treatments, displayed as heatmap (4569 total genes). **c** Heatmap of all 2003 differentially expressed genes commonly modulated by digitoxin treatment and gene therapy. Digitoxin-treated and control IB3–1 cell samples are shown along with gene therapy S9 cells. Vertical colorbars on the left-hand axis indicate directional expression groups: up-regulated by digitoxin treatment but down-regulated by gene therapy (purple, 476 genes), up-regulated by both conditions (red, 410 genes), down-regulated by both conditions (blue, 611 genes), and up-regulated by gene therapy but down-regulated by digitoxin (green, 506 genes)

To further understand differential drug effects, we measured genome-wide transcript abundances via mRNA sequencing and tested for differentially expressed genes (DEGs) in CF cells following digitoxin, gene therapy, and VX-drug treatments. Among all treatment comparisons, we detected 9750 unique DEGs and observed a major separation between digitoxin-treated and non-digitoxin-treated samples, regardless of other treatments (Fig. 5a, left-most groups with 10 nM digitoxin; right-most without). Digitoxin treatment alone induced differential expression of 7230 genes in IB3–1 cells compared to untreated controls. Transcriptome changes in gene therapy-treated S9 cells divides digitoxin from non-digitoxin-treated IB3–1 cells. TNFα, VX drugs, or gene therapy treatment without digitoxin collectively induced differential expression of 4569 genes compared to IB3–1 controls (Fig. 5b). Among these, gene therapy altered the expression of 3134 genes, while VX-770, VX-809, and VX-661 monotherapies induced changes in 1468, 104, and 3 genes, respectively. Of the genes significantly modulated by VX-770, 83% were uniquely up-regulated and 78% were uniquely down-regulated when compared to genes up and down-regulated by gene therapy. Thus, while VX-770 significantly altered the expression levels of a substantial number of genes, these changes were mostly in the opposite direction from those of gene therapy. In addition, VX-661 and VX-809 treatments globally altered substantially fewer genes compared to VX-770 and gene therapy.

We next examined functional gene ontology (GO) themes enriched in the genes significantly altered by the various treatment combinations. As shown in Table 1A, stable expression of the AAV-[*wildtype*]CFTR gene in CF cells resulted in elevation of genes associated with steroid biosynthesis, neurogenesis and cell development, and morphogenesis of epithelium, including gland and urogenital development. Gene therapy also caused *reduction* of genes associated with gene ontology themes for cytokine responses, Type 1 interferon signaling, mucin metabolism, and DNA/chromatin interactions (Table 1A). Many of these are clinically familiar functional themes for which elevation or reduction might be expected to flow from successful CF gene therapy. As shown in Table 1B, digitoxin monotherapy up-regulates genes associated with myeloid cell activation, ATP and NAD metabolic processes, and negative regulation of cell death. Themes down-regulated by digitoxin monotherapy included protein targeting to the endoplasmic reticulum and co-translational protein targeting. Finally, we examined functional themes for mono and combination VX drug treatments **(**Additional file 6)**.** VX-770, which induced > 1000 DEGs, was associated with up-regulated functional themes for response to unfolded protein and sterol metabolism. However, the ensemble of all DEGs down-regulated by VX-770 was not significantly enriched for GO processes. Genes up-regulated by VX-809 were also not significantly enriched for functional themes, although genes down-regulated by this drug included a small set of < 20 genes enriched for immune, inflammatory, and interferon signaling processes. VX-661 altered too few genes to find enriched ontologies.

**Table 1:**
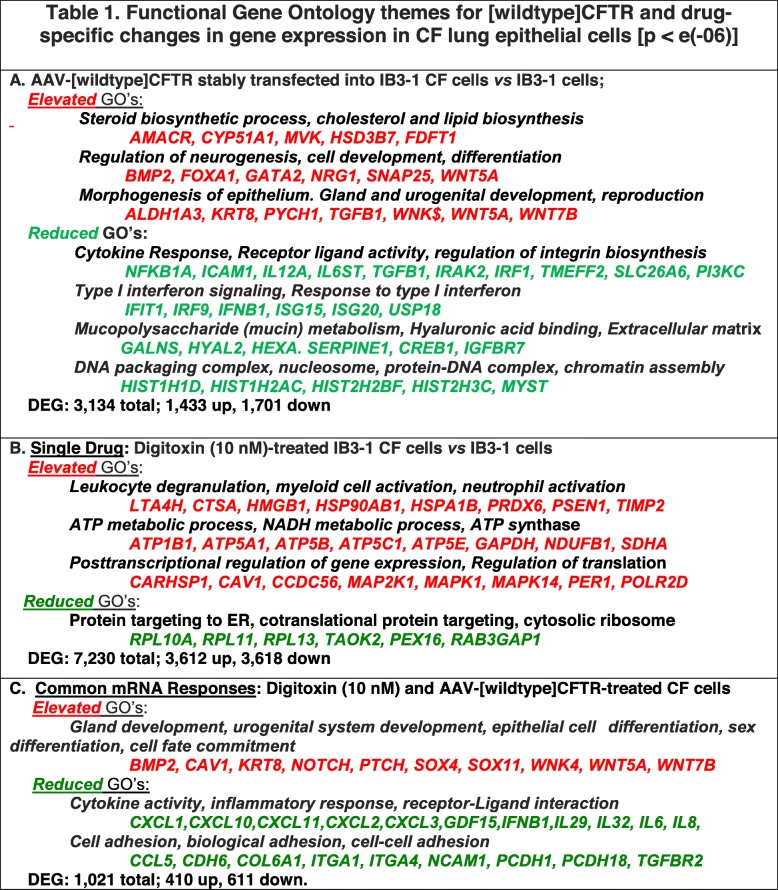
Functional Gene Ontology themes for [wildtype]CFTR and drug-specific changes in gene expression in CF lung epithelial cells [p < e(− 06)]

### Shared transcriptional responses between digitoxin treatment and gene therapy

We next searched our RNA-seq results for specific sets of genes whose expression was significantly altered by both digitoxin treatment and gene therapy. Consistently, this  analysis identified a significant set of 2003 genes altered by both treatments compared to IB3–1 controls (*p* < 4.7e-17, hypergeometric test) (Fig. 5c). Among these, 410 genes were *elevated* by both digitoxin treatment and gene therapy (red vertical bar on the left axis; also see Additional file 7**)** and 611 were *reduced* by both (blue vertical bar on left axis; also see Additional file 8). Of these, the number of genes down-regulated by both conditions (611) is more than expected by chance (*p* < 4.7e-12, hypergeometric test). Of the 3134 genes significantly affected by gene therapy, 1021 of these (32.6%) are also modified by digitoxin treatment and change in the same direction (see Table 1C). Among these consistently modified genes, morphogenesis of epithelium, gland and urogenital development, and reproduction are among the gene ontology themes significantly *enriched* in commonly elevated genes. The latter themes serve as a reminder that it is still poorly understood why CF males are functionally and structurally sterile [41, 42]. Among commonly *reduced* genes are enrichments for cytokine responses, receptor ligand activities, and integrin biosynthesis. This theme is characterized by *reduced* expression of component mRNAs for proinflammatory NFκB signaling, including *NFKB1*, the DNA binding subunit of the NFκB protein complex. In addition, cell-cell adhesion/fibrosis themes are enriched in the commonly *reduced* gene set. These shared themes suggest that digitoxin alone might be able to emulate some disease-relevant elements of gene therapy.

**Fig. 6:**
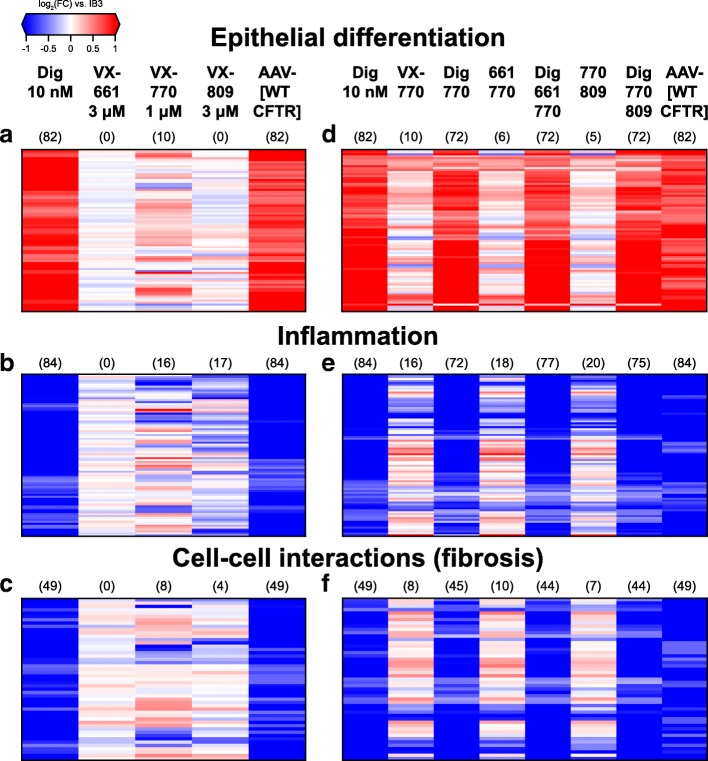
Common transcriptional changes induced by digitoxin and gene therapy involved in specific biological processes. Heatmaps of genes significantly over and under expressed (FDR < 0.01, |log_2_ fold-change| > 0.5, mean TPM > 1) by digitoxin and by gene therapy and enriched for select gene ontology categories: epithelial differentiation (panels **a** and **d**), inflammation (i.e. fibrosis, panels **b** and **e**), and cell-cell interaction (fibrosis) (panels **c** and **f**). The values displayed are expression log_2_ fold-changes for the labelled conditions against control IB3–1 cells; red indicates positive changes (i.e. overexpression), blue negative changes (underexpression), and white (neutral). In addition, the parenthetical numbers above the conditions indicate the numbers of genes found to be significant in individual pairwise analyses of the given treatment versus IB3–1 controls.

Among the 410 genes *elevated* by both digitoxin treatment and gene therapy, there is an 82 gene signature defined by ontologies for (in order of significance) glandular development, urogenital system development, epithelial cell differentiation, sex differentiation and cell fate commitment (Fig. 6a and Additional file 9**).** Hereafter we refer to this theme simply as epithelial differentiation. This gene expression signature includes mRNAs for *BMP2, CAV1, KRT8, NOTCH, PTCH1, WNK4, WNT5A*, and *WNT7B*. Many of these genes, including *KRT8*, have been explicitly associated with lung epithelial cell development from induced pluripotent stem cells [43], and they cluster together in Fig. 5c. KRT8 protein expression in IB3–1 cells is also significantly *increased* by digitoxin treatment (Additional file 10). VX-770 significantly up-regulates 7 of these 82 genes, while down-regulating 3. By contrast, neither VX-661 nor VX-809 significantly alter the expression of any genes in this group. Importantly, these increased functional distinctions were not apparent until we searched for overlaps between digitoxin treatment and gene therapy.

Among the 611 genes *reduced* by both digitoxin treatment and gene therapy, we identified two major functional themes: an inflammatory signature, defined by 84 genes enriched for cytokine activity, inflammatory response and receptor-ligand interactions ontologies (Fig. 6b and Additional file 11), and a fibrotic signature, which is defined by 49 genes enriched for cell adhesion, biological adhesion and cell-cell adhesion ontologies (Fig. 6e and Additional file 12). The 84 gene inflammatory signature included mRNAs for *IL-8*, *IL- 6*, *CCL5*, *CCL2*, *RELB*, *NFKB2*, *IKBKB*, and *CXCL1*. By comparison, VX-809 and VX-770 mono- treatments significantly reduced 17 and 12 of these 84 genes, respectively, with VX-770 elevating an additional four of these genes. By contrast, VX-661 did not significantly alter the expression levels of any of the genes in this set. Included in the fibrotic signature are mRNAs for *TGFBR2*, which encodes the principal receptor for TGFβ, and the collagen genes *COL6A1* and *COL6A2*. TGFBR2 protein expression in IB3–1 cells is also significantly *decreased* by digitoxin treatment (Additional file 10). As was the case for the overlapping up-regulated gene set, these decreased functional processes were not apparent until we searched for overlaps between digitoxin treatment and gene therapy.

Finally, to further test for potential interactions between digitoxin and the licensed VX drugs, we treated IB3–1 cells with VX drug combinations in the presence of digitoxin and assessed transcriptional changes in the genes defining these 3 signatures (Fig. 6d-f and Additional Files 13-15). We found that adjuvant digitoxin treatment induced transcriptional responses mirroring those observed in the digitoxin alone and gene therapy groups. Thus, adjuvant digitoxin can sustain its ability to emulate gene therapy in the presence of licensed VX drugs. We conclude that tested VX-drugs do not appear to interfere with digitoxin‘s ability to emulate disease-specific therapeutic aspects of gene therapy in CF lung epithelial cells.

### Comparison of in vitro and in vivo transcriptional responses to digitoxin treatment

**Fig. 7:**
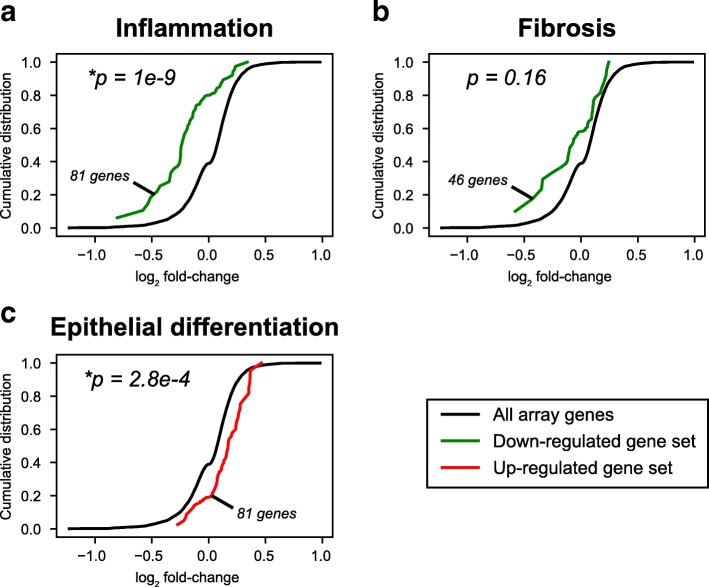
Gene set analyses of nasal epithelial cell mRNA expression from digitoxin-treated CF patients. (**a**) Pro-inflammatory gene signature. 81 of 84 genes in the *in vitro* digitoxin/gene therapy-dependent gene signature for inflammation found in the in vivo affymetrix array. (**b**) Pro-fibrosis gene signature. 46 of 49 genes in the *in vitro* digitoxin/gene therapy-dependent gene signature for fibrosis found in the *in vivo* affymetrix array. (**c**) Epithelial differentiation signature. 81 of 82 genes in the in vitro digitoxin/gene therapy-dependent gene signature for epithelial differentiation found in the *in vivo* affymetrix array. Data and color-coding: Post- vs. pre-treatment Affymetrix microarray mRNA expression log2 fold- changes of nasal epithelial cell biopsies from CF patients treated with 0.1 mg digitoxin/day for 28 days. Average log2 fold-changes for all array genes (black lines) and sub-sets of genes defining three overlapping digitoxin/gene therapy gene sets identified from* in vitro *RNA-seq data (colored lines; green for down-regulated sets and red for up-regulated sets) are shown. Numbers of genes present on array in defined gene sets are displayed, along with two-tailed Mann-Whitney-Wilcoxon U-test *p*-values for set genes versus all genes. * indicates *p* < 0.05

To test whether our in vitro transcriptional data of cultured digitoxin-treated CF cells had any clinical relevance for CF patients, we interrogated Affymetrix exon array mRNA expression data collected from nasal biopsies of digitoxin-treated CF patients [35]. We calculated expression fold changes between pre- and post-treatment for 8 CF patients, 4 of whom were homozygous and 4 of whom were heterozygous for [*F508de*l]CFTR, who had received a conventional 0.1 mg digitoxin dose for 28 days. We tested for consistent fold-change trends in genes defining the three in vitro expression signatures coincidently modulated by digitoxin treatment and gene therapy. From these aggregate views of the patient expression data, we detected a significant global shift towards reduction of genes in the inflammatory set (Fig. 7a, *p* = 1e-9) and a significant shift towards *elevation* in the epithelial differentiation set (Fig. 7c, *p* = 2.8e-4). Genes in the fibrosis set trended towards down-regulation, but the overall gene set shift was not significant (Fig. 7b, *p*= 0.16). Digitoxin dependent changes can be seen in Additional file 16 (inflammation), Additional file 17 (fibrosis) and Additional file 18 (epithelial differentiation). Thus, the effects of digitoxin on parental CF lung epithelial cells significantly parallel the effects of digitoxin on CF patient nasal epithelial cells when viewed from the perspective of functional gene ontology themes similarly shared by digitoxin and AAV-[*wildtype*]CFTR gene therapy. We conclude that studies with digitoxin on the CF IB3–1 lung epithelial cell system appear to be clinically associative for mRNA expression in upper airway epithelial cells from CF patients.

## Discussion

The need for anti-inflammatory drugs as adjuvants to standard CF therapy has been prominent in the CF literature for decades, and remains a challenge even in this time of licensed corrector and potentiator drugs [17, 25–28]. Here, we show that treatments with clinically relevant concentrations of individual or combination corrector or potentiator drugs are relatively ineffective at suppressing secretion of IL-8, IL-6 and GM-CSF proteins from cultured CF lung epithelial cells. By contrast, additions of digitoxin, alone or in combination with corrector/potentiator drugs, potently suppresses not only IκBα and NFκB activation, but also baseline and TNFα-activated secretion of these three proinflammatory mediators. Importantly, transcriptional studies via RNA-seq strongly support these proteomic results. In addition, we find that the transcriptomes of gene therapy-treated and digitoxin-treated CF cells substantially overlap. Among genes that are significantly modified in parallel by these two treatments, we identified an 84 gene signature for *reduced* inflammation, a 49 gene signature for *reduced* fibrosis, and an 82 gene signature for *elevated* epithelial differentiation. Consistently, digitoxin also *suppressed* expression of TGFBR2 protein, the receptor for TGFβ, the master regulator of fibrosis [44]. Digitoxin also *increased* expression of KRT8 protein, a biomarker for epithelial differentiation [43]. Thus, while digitoxin does not affect trafficking of mutant CFTR, and only modestly opens the [G551D]CFTR channel to potentiate chloride transport across the membrane, this drug is capable of emulating other physiologically important corrections expected from gene therapy. Specifically, these corrections include reduced inflammation and fibrosis and increased epithelial differentiation. By contrast, our transcriptional data indicate that the licensed VX drugs minimally impact these shared gene therapy/digitoxin signatures. Finally, using the biological features uncovered in the in vitro data, we found a consistent relationship with clinically derived in vivo mRNA expression data from nasal epithelial biopsies of digitoxin-treated CF patients. Importantly, this insight was not apparent when the results of our clinical trial were first published [35]. We therefore suggest that using digitoxin to treat CF, either alone or as an adjuvant drug, is worthy of further consideration.

Insight into the mechanism for these shared functional gene ontology themes may be gained by consideration of early studies showing that digitoxin blocks the interaction between the TNFα/TNFR1 complex and TRADD [33, 34]. TRADD is the first intracellular adaptor for TNFα signaling to activate NFκB, and therefore downstream *IL-8* expression. We have previously shown that high levels of *TRADD* mRNA characterize epithelial brush biopsies of CF patients with the most severe disease, as defined by the rate of FEV1 decline [45]. More recently, we reported that [wildtype]CFTR also directly interacts with TRADD and tonically directs TRADD for proteosomal destruction [14]. By contrast, we showed in that report that the mutants [*F508del*] and [G551D]CFTR proteins fail to bind TRADD, and thus fail to inhibit downstream activation of NFκB. These two different experiments thus indicate that TRADD is a common inflammation-controlling target for both digitoxin and [wildtype]CFTR. It is thus possible that coincident interactions with TRADD may be the basis for the large scale and highly functional transcriptional overlap between digitoxin and gene therapy. Digitoxin thus differs from other candidate NFκB inhibitors with different mechanisms of action in this way, whose renal and CNS side effects are said to have rendered them generally useless for clinical applications [46]. Finally, it is possible that digitoxin may also inhibit pro-fibrotic signaling through its action on TRADD. For example, increased fibrosis is also known to be a direct consequence of TNFα-driven activation of TGFβ1 signaling [47–49]. Thus, by blocking expression of both *TGFBR2* mRNA and TGFBR2 protein, the receptor for TGFβ, both digitoxin and gene therapy suppress profibrotic TGFβ signaling. Hence, these data suggest that digitoxin appears to uniquely address the root cause of not only inflammation but also fibrosis in CF.

Elevation of mRNAs associated with epithelial differentiation may also be related to TRADD function. It is possible that the effect of digitoxin and gene therapy on CF cells is to start repair of developmental defects in these cells, and thus reset the differentiated state of the cells. In fact, many of the mRNAs in this category are directly associated with regulating differentiation of stem cells into the more differentiated pseudostratified epithelium lining the airway. Here, the action of digitoxin and gene therapy on TRADD function may provide a clue. In the lung, Activin A, a member of the TGFβ superfamily, initiates the differentiation process from iPSCs (induced pluripotent stem cells) to form definitive endoderm [50]. To convert definitive endoderm to anterior foregut endoderm, these cells must be incubated in NOGGIN to *reduce* levels of TGFβ, and inhibit BMP signaling. In as much as there is significant reduction in *TGFBR2* due to digitoxin blocking TRADD-dependent activation of NFκB, TGFβ signaling would be suppressed, independent of TGFβ availability. Both digitoxin and gene therapy also elevate *KRT8*, a biomarker for conversion of basal stem cells into differentiated epithelial cells, as well as several NOTCH and WNT isoforms. Relevantly, following differentiation of stem cells into basal stem cells in the lung, NOTCH signaling takes over as a driver for the more differentiated pseudostratified epithelial cells lining the lung. We therefore suggest that this uniquely elevated functional gene ontology theme may reflect the action of digitoxin or gene therapy to reset and repair CF related defects in the differentiated state of CF cells. This polypharmacy property, on the one hand anti-inflammatory and anti-fibrotic, and on the other pro-differentiation, may uniquely distinguish digitoxin from other candidate drugs being developed for CF.

Finally, to our knowledge these are the first data to characterize changes in mRNA expression caused by licensed CF correctors and potentiators, including VX-770 (Ivacaftor®), [*VX-770/VX-809*] (Orkambi®), and [*VX-770/VX-661*] (Symdeko®). Based on the ELISA and RNA-seq data, *reduction* of TNFα/NFκB-driven inflammation is not among the significant functional gene ontology themes for the VX drugs (see Additional file 6). While mono-therapy with VX-809 alone can reduce expression of *IL-8* mRNA, it does not significantly affect IL-8 protein expression. However, mixing VX-809 with VX-770 results in loss of this effect on *IL-8* mRNA. This result is reminiscent of previous reports of interference between these two drugs [36]. In addition, none of the VX drugs, alone or in combination, affect IL-6 mRNA or protein expression. Furthermore, none of the VX drugs are characterized by the digitoxin-defined gene ontology themes for *reduced* fibrosis or elevated epithelial differentiation. Thus, while these VX drugs may sufficiently modify folding of mutant CFTR to enable correction of trafficking or potentiation of chloride conductance, the drug-dependent modifications in folding are clearly not sufficient to correct other functional deficits. Here digitoxin may make a significant contribution as an adjuvant CF drug, since it addresses the root causes of multiple disease-related dysfunctions in CF.

In conclusion, the motivation for having tested the small molecule digitoxin in these ways has rested on our earlier discovery of its ability to suppress IL-8 secretion from CF lung epithelial cells [33, 34], and more recently on its suppressive effects on *IL-8*, *IL-6* and related mRNAs in CF patients [35]. Based on a new analysis of the clinical trial data we suggest that either independent use of digitoxin, or adjuvant use with the licensed VX drugs, may confer reduced signaling for inflammation and fibrosis and increased signaling for epithelial differentiation.

## Conclusions

1. Cystic Fibrosis lung epithelial cells exposed to digitoxin exhibit significant suppression of both TNFα/NFκB signaling and downstream secretion of IL-8, IL-6 and GM-CSF proteins, whether in the presence or absence of licensed corrector or potentiator drugs.
2. Transcriptomes of drug-treated or gene therapy-treated CF cells indicate that only digitoxin significantly emulates gene therapy regarding gene expression signatures for reduced inflammation and fibrosis, as well as elevated epithelial differentiation
3. Transcriptional responses shared by gene therapy and digitoxin treatment in vitro are consistent with in vivo expression changes from nasal epithelial biopsies of digitoxin-treated CF patients.
4. Digitoxin may contribute to enhancing the efficacy of currently licensed correctors and potentiators in CF patients.

## Additional Files

**Additional file 1.** Materials and Methods.

**Additional file 2.** Abbreviations.

**Additional file 3.** Influence of digitoxin and individual VX drugs on cytokine and chemokine secretion from CF IB3–1 cells.

**Additional file 4.** Influence of digitoxin and multiple VX drugs on cytokine and chemokine secretion from CF IB3–1 cells.

**Additional file 5.** Influence of digitoxin and VX-770 on chloride transport by FRT cells with the [G551D]CFTR mutation.

**Additional file 6.** Functional Gene Ontology themes for [wildtype]CFTR Gene Therapy, and Drug-specific changes in CF lung Epithelial cells.

**Additional file 7.** Genes up-regulated by both digitoxin treatment and gene therapy.

**Additional file 8.** Genes down-regulated by both digitoxin treatment and gene therapy.

**Additional file 9.** Genes upregulated by digitoxin treatment and gene therapy against IB3-1 controls annotated to epithelial differentiation ontologies, and compared in parallel to effects of VX-661, VX-770 and VX-809.

**Additional file 10.** Digitoxin-dependent changes in protein expression for IL-8, IL-6, TGFBR2 and KRT8.

**Additional file 11.** Genes downregulated by digitoxin treatment and gene therapy against IB3-1 controls annotated to inflammatory ontologies, compared in parallel to effects of VX-661, VX-770 and VX-809.

**Additional file 12.** Genes down-regulated by digitoxin treatment and gene therapy against IB3-1 controls annotated to cell-cell interaction/fibrosis ontologies, and compared in parallel with VX-661, VX-770 and VX-809.

**Additional file 13.** Effects of digitoxin on licensed VX-drug and drug combinations on reduced expression of mRNAs common to digitoxin and AAV-[wildtype]CFTR for the functional GO theme of inflammation.

**Additional file 14.** Effects of digitoxin on licensed VX-drug and drug combinations on reduced expression of mRNAs common to digitoxin and AAV-[wildtype]CFTR for the functional GO theme of cell-cell interactions/fibrosis.

**Additional file 15.** Effects of digitoxin on licensed VX-drug and drug combinations on increased expression of genes common to digitoxin and AAV-[wildtype]CFTR for the functional theme of epithelial differentiation.

**Additional file 16.** Comparison of genes down-regulated by digitoxin treatment and gene therapy in IB3-1 cells compared to controls by RNA-seq and annotated to inflammatory processes versus Affymetrix log2 expression fold-changes from CF patoents treated with 0.1 mg daily digitoxin for 28 days (pre-treatment vs post-treatment, Zeitlin et al, 2017).

**Additional file 17.** Comparison of genes down-regulated by digitoxin and gene therapy in IB3-1 cells compared to controls by RNA-seq and annotated to fibrotic processes versus Affymetrix log2 expression fold-changes from CF patients treated with 0.1 mg daily digitoxin for 28 days (pre-treatment vs post treatment, Zeitlin et al, 2017).

**Additional file 18.** Comparison of genes up-regulated by digitoxin treatment and gene therapy in IB3-1 cells compared to controls by RNA-seq and annotated to epithelial differentiation processes versus Affymetrix log2 expression fold-changes from CF patients treated with 0.1 mg daily digitoxin for 28 days (pre-treatment vs post-treatment, Zeitlin et al, 2017).

## Data Availability

The datasets generated and/or analysed during the current study will be available in the Gene Expression Omnibus (GEO) database (https://www.ncbi.nlm.nih.gov/geo/), sponsored by the National Institutes of Health (NIH), Bethesda, MD, USA. Patient-derived mRNA expression data used in this paper are available from the Gene Expression Omnibus database under accession number GSE76347.

## Abbreviations

AAV-[*wildtype]CFTR*: AAV is Adeno-Associated-Virus, which is the gene therapy vector carrying the [wildtype]CFTR into the IB3–1 cell. The transfection in this system is permanent
DNAse I: This enzyme is licensed as Pulmozyme®
ELISA: Enzyme-linked immunosorbent assay
*[F508del*]CFTR: The principal cystic fibrosis mutation in which phenylalanine at amino acid position 508 is deleted. This mutation is also written as [F508del]CFTR, [Phe508del] CFTR, and [c.1521_1523delCTT]CFTR
**[***G551D*]CFTR: The less frequent cystic fibrosis mutation in which glycine at amino acid position 551 is replaced by aspartic acid. This mutation is also written as [c.1652G > A]CFTR; and [p.Gly551Asp]CFTR
GM-CSF: Granulocyte Monocyte Colony Stimulating Factor functions as a cytokine which stimulates stem cells to produce granulocytes, including neutrophils and monocytes
IB3–1 cell: IB3–1 cells are cultured cells from the diseased lung of a CF lung transplant patient at Johns Hopkins University whose genotype was *[ΔF508*/*W1282X*]. Since the mRNA for [W1282X]CFTR is unstable, the genotype for IB3–1 cells is operationally just [ΔF508]. Gentamycin is omitted from LHC-8 medium to preclude read-through. The CFTR genes in these cells are run by a native promoter. To the best of our knowledge, “IB3–1” stands for “infected bronchus #3, 1st incubator
IB3–1/S9 cell: These are daughter IB3–1 cells which have been permanently transfected by AAV- [wildtype]CFTR
IκBα: IkappaBalpha stands for inhibitor of kB α.
IL-6: Interleukin-6, a proinflammatory cytokine which also stimulates autoimmune processes
IL-8: Interleukin-8, is the most potent and efficacious known chemokine (chemo-attractant) for neutrophils. High levels of IL-8 are found in the CF airway following secretion by CF airway epithelial cells. IL-8 is thought to be the biological basis for high levels of neutrophils in the CF lung. IL-8 also functions as an angiogenic factor by promoting growth of blood vessels, and is used by cancer cells in tumors for this purpose
LHC-8 medium: “LHC-8” stands for the 8th medium, developed for growth of lung epithelial cells by the NIH/NCI Laboratory of Human Carcinogenesis
NFκB: Nuclear factor kappa light chain enhancer of activated B cells.
RNASeq: A next generation method for quantitatively and comprehensively measuring mRNAs in biological samples. The method is based on “sequence by synthesis” in which the exact sequence of cDNAs, copied from a collection of pure mRNAs, ensures identify and specificity
VX-661 + VX-770: A licensed combination drug from Vertex, called Symdeko®, for CF patients both homozygous and heterozygous for the *[F508del*]CFTR mutation
VX-661: A candidate drug from Vertex, termed Tezacaftor®, that functions as a corrector for the *[DF508*]CFTR trafficking mutant
VX-770 + VX-809: A licensed combination drug from Vertex, called Orkambi® to treat the common *[F508del*]CFTR mutation
VX-770: A drug from Vertex, licensed as Ivacaftor®, that induces chloride channel activity in the [*G551D*]CFTR gating mutant
VX-809: A drug from Vertex, licensed as (Lumacaftor®), that corrects the trafficking defect in the *[F508del*]CFTR trafficking mutant
